# A Multi-Component Model of the Developing Retinocollicular Pathway Incorporating Axonal and Synaptic Growth

**DOI:** 10.1371/journal.pcbi.1000600

**Published:** 2009-12-11

**Authors:** Keith B. Godfrey, Stephen J. Eglen, Nicholas V. Swindale

**Affiliations:** 1 Department of Applied Mathematics and Theoretical Physics, University of Cambridge, Cambridge, United Kingdom; 2 Department of Ophthalmology and Visual Sciences, University of British Columbia, Vancouver, Canada; Indiana University, United States of America

## Abstract

During development, neurons extend axons to different brain areas and produce stereotypical patterns of connections. The mechanisms underlying this process have been intensively studied in the visual system, where retinal neurons form retinotopic maps in the thalamus and superior colliculus. The mechanisms active in map formation include molecular guidance cues, trophic factor release, spontaneous neural activity, spike-timing dependent plasticity (STDP), synapse creation and retraction, and axon growth, branching and retraction. To investigate how these mechanisms interact, a multi-component model of the developing retinocollicular pathway was produced based on phenomenological approximations of each of these mechanisms. Core assumptions of the model were that the probabilities of axonal branching and synaptic growth are highest where the combined influences of chemoaffinity and trophic factor cues are highest, and that activity-dependent release of trophic factors acts to stabilize synapses. Based on these behaviors, model axons produced morphologically realistic growth patterns and projected to retinotopically correct locations in the colliculus. Findings of the model include that STDP, gradient detection by axonal growth cones and lateral connectivity among collicular neurons were not necessary for refinement, and that the instructive cues for axonal growth appear to be mediated first by molecular guidance and then by neural activity. Although complex, the model appears to be insensitive to variations in how the component developmental mechanisms are implemented. Activity, molecular guidance and the growth and retraction of axons and synapses are common features of neural development, and the findings of this study may have relevance beyond organization in the retinocollicular pathway.

## Author Summary

Neural development is a process that involves a wide range of behaviors. As a result of these behaviors, neurons are able to extend axons to different brain areas and produce stereotypical patterns of innervation. One of the most commonly studied of these projections is in the visual system, where retinal axons project to multiple brain regions and produce retinotopic maps. This study examines the relative roles and interactions of different neural mechanisms in guiding axon growth and generating retinotopic order. We did this by producing a computational model of retinotopic development that represented many of the neural mechanisms thought to be involved, including axon and synapse growth, molecular guidance and synapse plasticity. Our results suggest that synaptic plasticity is realized by variation in the number of synapses between neurons, not through alteration of individual synaptic weights; that lateral connectivity between collicular neurons is not required for organization; and that axon arbor development does not require the gradient tracking abilities of growth cones. The mechanisms underlying neuronal development in the visual system are also observed in many other brain areas, so the findings here should apply more generally.

## Introduction

During neural system development, groups of neurons project to various areas of the brain and produce stereotypical patterns of innervation. These organization patterns are an emergent property of the physiological mechanisms regulating neural behavior. In the visual system these mechanisms include molecular guidance [1],[2], spontaneous correlated activity in the form of retinal waves [3]–[5], neurotrophic factor release and uptake [6], spike-timing-dependent plasticity (STDP) [7],[8] as well as the growth and retraction of axons and synapses. Similar phenomena are observed in many other brain areas during development [9]–[13]. An important question is how these underlying phenomena combine to produce the emergent patterns of connections seen throughout the brain. A well studied example of such organization is the retinotopically ordered projection from the retina to the thalamus and superior colliculus.

Many computational models have examined how one or more of these phenomena are able to produce retinotopic organization (e.g., [14]–[21]). So far, however, none of the models has brought together this diverse set of physiological behaviors, and only a few (e.g., [22]) have addressed development from the perspective of individual axons and how they can grow, branch and retract to reach their retinotopically correct termination zones. Framing development from this perspective is important, as neural connection patterns are ultimately the result of axon growth and branching, hence constraining a model by the physical and geometrical constraints of the axon is a prerequisite to understanding how projections form. An axon extending through any neuropil consisting of cells, axons and dendrites is analogous to a rope being pulled through a corn field: once the rope is extended, lateral motion is not possible without knocking over corn stalks [23]. Similarly, an axon has very restricted lateral motion once it has extended and branched throughout the neuropil. To explain neural organization such as retinotopic development, a model needs to describe not only how these physiological behaviors contribute to development, but also how observed patterns of development can be achieved in light of the physical constraints placed on axon movement.

**Figure 1:**
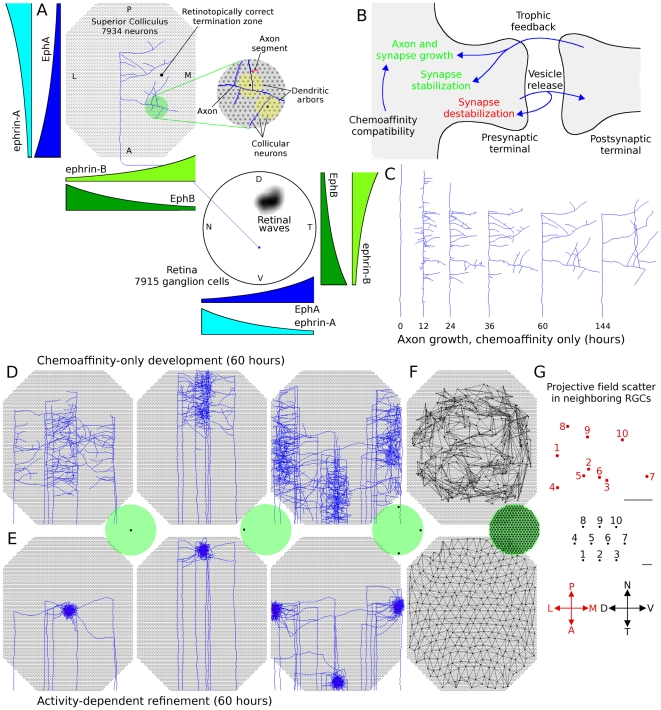
Model layout and axon arbor refinement. *A*. Cartoon showing simulated retina and colliculus. RGCs extended into the colliculus and arborized there. Axon growth was mediated by molecular gradients and trophic feedback to synapses. Retinal waves drove patterns of RGC spiking. Each axon was composed of a series of axon segments (an example shown in red in the expanded view) and each segment was able to produce synapses with overlapping dendrites (yellow). *B*. Axon and synapse growth was enhanced by chemoaffinity compatibility and trophic feedback. Trophic factor stabilized synapses, while use destabilized them. Axons retracted in areas of the arbor with lower relative chemoaffinity and trophic feedback. *C*. Time-lapse development of the axon shown in *A*. This and most other visually observed axons achieved the majority of their growth in 48–60 hours. *D*. Axon arbors from groups of neighboring RGCs from different retinal locations after chemoaffinity-driven development. A miniature retina is shown in green and the black dots represent the location where the axons originate from. Axons produce a coarse retinotopic projection. *E*. The same axon arbors shown in *D* after 60 additional hours of growth, mediated by both chemoaffinity and activity-dependent trophic feedback. *F*. Map of the projection of individual RGCs after chemoaffinity mediated growth (top) and activity-dependent refinement (bottom). *G*. Projective field (PF) scatter of neighboring RGCs in a refined retinotopic projection. The PF center (red) from 10 neighboring RGCs (black) from a randomly selected retinal location. While global topography is observed in the refined retinal projection (*F*), at the local level the order is less regular. Scale bars 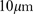.

This study presents a model of retinocollicular development that combines phenomenological approximations of the aforementioned physiological behaviors and examines how these can guide the extension, branching and retraction of individual axons in such a way that leads to a refined arborization at the retinotopically correct location in the colliculus. The stages of development follow those previously described for mouse and chick [24]. In summary, retinal axons enter the anterior side of the colliculus and extend in a largely linear manner to the posterior side. Interstitial branches then sprout and extend towards the retinotopically correct area of the colliculus for the given axon, based on chemoaffinity compatibility between each axon and the expression of molecular markers in the colliculus (Fig. 1*A*). Activity-dependent trophic feedback mediates growth and retraction of individual synapses, with trophic factor stabilizing synapses that contribute to spiking activity in the postsynaptic neurons and synapses that receive insufficient trophic feedback retracting (Fig. 1*B*). Correlated retinal activity, in the form of retinal waves, provides spatial information allowing synapses from retinal ganglion cells (RGCs) originating from near the same point in the retina to stabilize on the same collicular neurons. Trophic factors enhance axon and synapse growth in the areas of the axon where they are received and STDP modulates the excitatory strength of individual synapses.

This study continues in the spirit of previous theoretical work on hybrid models (e.g., [25]), allowing the relative roles of and interactions between these different physiological behaviors to be studied, and has generated several new findings. Most significantly, retinotopic organization and refinement appears to be a stable emergent property of the core assumptions so long as the functional behaviors of retinal waves, molecular guidance cues and activity-dependent trophic factor release were represented in the model. The characteristic of retinal waves that was important was the overall correlational structure of activity and not the specific spatiotemporal properties of the waves. Alteration in the correlational structure by using simulated retinal activity similar to that observed in the 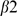 mutant mouse [26] disrupted the ability of axons from neighboring RGCs to produce overlapping arbors in the colliculus. Neither STDP nor any form of plasticity occurring at the level of individual synapses was necessary for refinement, and analysis of the model suggests that Hebbian synaptic plasticity is a slow-acting process that is instead realized by the addition and subtraction of synapses. Gradient detection by axon growth cones was not required to achieve retinotopic organization or refinement once axons had reached the colliculus, as each axon was able to guide growth based on gradient differentials across its arbor.

## Results

The model addresses retinocollicular development over five days (120 hours) of simulated time, similar to the one week period of maturation of the retinocollicular (retinotectal) projection in mice and chicks [24],[27]. While molecular guidance cues and retinal waves are both present throughout this age, modeled development occurred in two stages, each lasting 60 hours. During the first stage of development, an axon's propensity for growth was mediated only by its chemoaffinity compatibility with surrounding tissue, while during the second stage trophic factor receipt by synapses on the axon also contributed to guide growth. Fig. 1*C* shows the development of a representative axon during chemoaffinity regulated growth. Fig. 1*D* shows the chemoaffinity-mediated axon growth from groups of neighboring retinal ganglion cells (RGCs) at five different retinal locations. A coarse topological organization is apparent.

**Figure 2:**
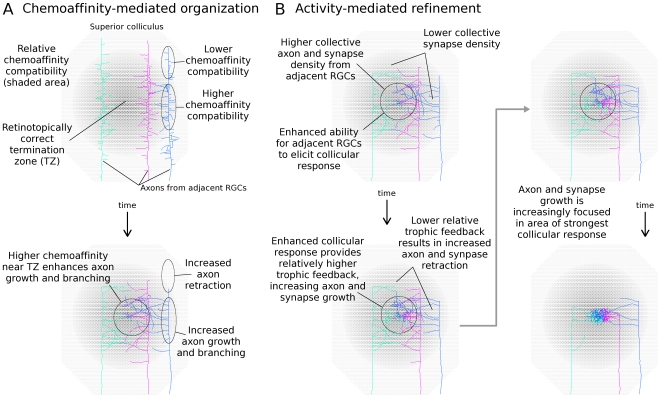
Overview of developmental mechanism. *A*. During chemoaffinity-mediated axon growth, axon branching occurs preferentially in areas of the arbor with increased chemoaffinity compatibility with nearby collicular neurons, and axon retraction is more likely in areas of reduced chemoaffinity compatibility. This mechanism results in each RGC axon extending and branching in the vicinity of the retinotopically correct termination zone (TZ). *B*. Nearby RGCs have similar molecular markers and hence similar TZs. While individual axons rarely reached their TZ, the collective axon projection from neighboring RGCs had a higher density near the TZ, and with higher axon density there was also higher synapse density. Because retinal waves cause nearby RGCs to simultaneously burst, the higher density of synapses near the TZ caused these collicular neurons to be more likely to respond to bursting activity of these RGCs. Increased collicular response led to increased trophic feedback to these synapses (Fig. 1*B*), resulting in enhanced synapse and axon growth in this area of increased synapse density. Synapses further from the TZ were less able to induce a spike and were thus more likely to retract. It is worth noting that individual synapses were weak and it typically took spikes in several RGCs to elicit a spike in a collicular neuron. Homeostatic mechanisms controlling each collicular neuron's activity level restricted the number of innervating synapses on over-active neurons and served as a normalizing force.

After 60 hours, trophic factors also contributed to guiding axon growth and synapse creation. Trophic factor was released by postsynaptic terminals where a presynaptic spike preceded a postsynaptic spike within tens of milliseconds, and was taken up by the presynaptic terminal. As described later, to achieve a smooth retinotopic mapping, it was necessary to delay activity-dependent trophic feedback to axon growth until axons had produced diffuse arbors in the retinotopically correct areas of the colliculus. Fig. 1*E* shows the continuation of development from Fig. 1*D* after activity-dependent mechanisms became active. An overview of how the mechanisms of the model generate retinotopic organization and refinement is shown in Fig. 2. In summary, molecular guidance cues guide axons to near their retinotopically correct areas of the colliculus. While individual axons arbors are only loosely targeted, nearby RGCs collectively produce arbors with highest density near the retinotopically correct termination zone. Axon density corresponds with synapse density, resulting in a enhanced collicular response in the areas of higher axon density. This increased response results in increased activity dependent feedback from these collicular neurons, increasing local axon and synapse growth and resulting in an increasingly refined arbor. A movie showing RGC axon development over the full 120 hours can be downloaded as supplementary material (Video S1).

**Figure 3:**
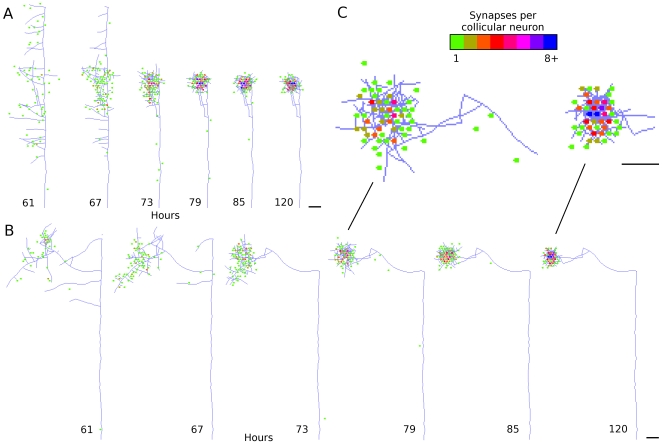
Individual axon arbor refinement. *A*,*B*. Axon development from two arbitrarily selected RGCs after the onset of activity-dependent feedback. Synapses are shown as dots, with the location of the dot representing the position of the target collicular neuron, and the color of the dot indicating the number of synapses between the RGC and each collicular neuron. The initially broad distribution of synapses and axon segments quickly refines. The majority of refinement is observed in the first 24 hours after trophic feedback begins to influence axon growth. *C*. Expansion of same arbor at two times during development. Scale bars 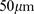.

Upon the onset of activity-dependent feedback, the diffuse, chemoaffinity guided arborizations quickly refined. Fig. 3*A,B* shows the development of two axons at six hour intervals after activity-dependent feedback began to influence axon growth. Initial synapse distribution from any particular axon was diffuse with most arbors becoming largely refined after 24 hours simulated time. Because of the relatively long duration of individual simulations (1–6 days, realtime), analysis of the model focused on its qualitative behavior and attempts were not made to tune the model in such a way as to achieve a particular quantitative goal, such as development time or receptive field size. Quantitative measures were made to help assess qualitative behavior. The average receptive field (RF) radius (see Methods) for individual collicular neurons was 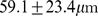 (n = 7935 collicular neurons) and the average projective field (PF) radius for RGCs was 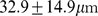 (n = 7914 RGCs). To assess the continuity of the retinal projections, the RF and PF of groups of neighboring neurons were also measured (19 adjacent RGCs or collicular neurons from 7279 and 7278 non-border locations in the retina and colliculus, respectively). The RF for groups of collicular neurons was 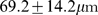 and the PF for groups of RGCs was 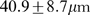, an increase of 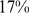 and 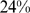 over individual RF and PF, respectively. This small increase in size for groups of cells versus individual cells indicates that there was considerable overlap in the RF and PF of groups of neighboring cells. Visual analysis of the projections confirmed this interpretation.

**Figure 4:**
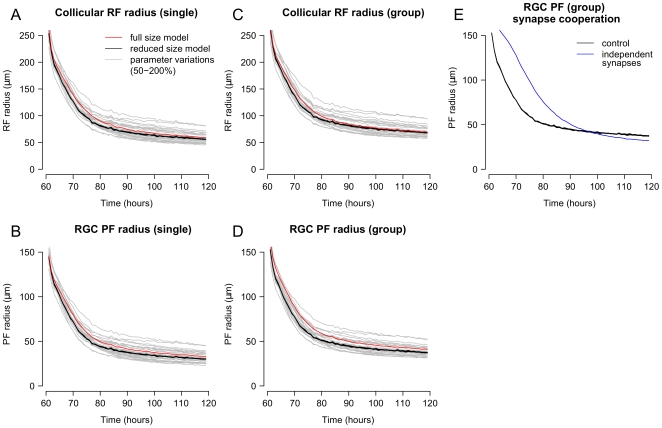
Refinement of retinal projection. *A*–*D*. Changes in the average receptive field (RF) and projective field (PF) with time both for individual neurons and groups of adjacent neurons. Comparison of full-size (red) and reduced size (black; n = 6) models show that refinement is quantitatively similar for different model sizes and shapes, and for multiple simulation runs. The effects of individually varying model free parameters (Table 1) from 50% to 200% of base values are shown in grey. Changes increased and decreased retinotopic refinement but the overall pattern of refinement remained similar. Parameter values in the model were not optimized. *E*. Modifying the model to use “independent” synapses, whose survival depended entirely on their own trophic factor receipt, instead of the default behavior where synapses helped to stabilize both themselves and their neighbors, changed the time-course of refinement but did not otherwise significantly affect it. These simulations were performed on the reduced size model.

In addition to the shape of the simulated retina and colliculus described above (Fig. 1), simulations were carried out on a reduced form of the model where a slice of the simulated retina (central 30% of D-V axis; 3023 RGCs) projected to a slice of collicular neurons (central 30% of L-M axis; 2695 collicular neurons; see Methods). The smaller model had qualitatively and quantitatively similar behavior as the full model (Fig. 4*A–D*, compare red and black traces). Simulation of the reduced size model was faster than the full model and analysis of the model's behavior was carried out using this smaller implementation. There was limited variability in the RF and PF radii between multiple simulations runs (n = 6). The individual RF for collicular neurons was 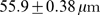 (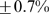) and the group RF was 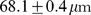 (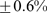). The average individual PF was 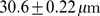 (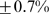) and the group PF was 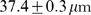 (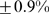). Because of the limited variability, and the duration of individual simulations, all quantitative measures of model behavior (e.g., parameter exploration and STDP analysis) were based on the behavior of the 3023 RGC or 2695 collicular neurons in the reduced size model, measured from a single simulation run, except as otherwise noted.

**Figure 5:**
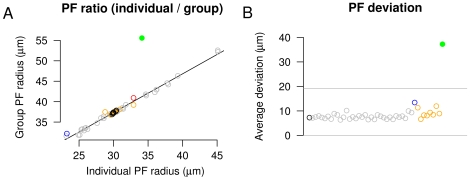
Stability of model in response to changes in parameters. *A*. The ratio of individual PF to group PF for all simulations shown in Figs. 4 and 6, displayed using the same colors (black control; grey parameter variation; orange retinal wave variations; green 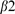 mutant; blue independent synapses; red full size model). For all model configurations using normal wave activity, there is a consistent relationship and similar size between the PF of individual and groups of RGCs, showing a large degree of overlap in the arbors of nearby RGCs. Significant alterations in the correlational structure of retinal activity (simulated 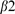 mutant, see Fig. 6) disrupts the regularity of axonal projections (note green outlier). *B*. Analysis of retinotopic order. The deviation of each RGC's axonal projection is compared to those occurring in a “normal” map. Average values for each simulation are shown, using colors as shown in *A*. The deviation averaged over most simulations runs was less than the distance between neighboring collicular neurons (10 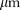), while others were slightly above this metric, showing that global order was maintained. Grey lines show 95% confidence intervals for control simulation. The single outlier (green) still demonstrated normal retinotopic order, as the average deviation of each RGC projection from normal was less than the distance between 4 collicular neurons (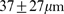). For comparison, disruption of global order through blocking chemoaffinity in the reduced size model resulted in a deviation of 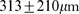.

The model was analyzed to evaluate its stability and to examine the effects of modifying the physiological behaviors on which it was based. To evaluate the stability of the model, each of 16 free parameters (Table 1) were individually altered from 50% to 200% of their baseline values and the retinotopic organization and refinement of the model was analyzed (Fig. 4*A–D*, grey traces). In all cases, development was qualitatively similar. The PF of individual RGCs was similar to the PF of groups of neighboring RGCs, indicating a large degree of overlap and limited scatter at the local level. The ratio of the PF size between individuals and groups of neighboring RGCs was consistent for all parameter variations (Fig. 5*A*, grey circles). Global retinotopic order was assessed by comparing the target position of RGCs between simulations. To do this, the average PF center of each RGC was calculated over five control simulations and this average was used to produce a “normal” map. RGC projections in parameter exploration simulations were then compared to the normal map, and the deviation of each RGC projection from normal was measured. The average deviation in these simulations was approximately the distance between adjacent collicular neurons (10 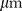), demonstrating that global order was maintained (Fig. 5*B*, grey circles).

**Table 1:** Model parameters.

Variable	Value	Range	Description
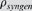	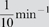	50%–200%	Base probability of synapse formation.
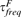	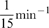	50%–200%	Time constant for estimating neural firing rate
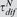	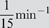	50%–200%	Rate of diffusion of trophic factor between connected axon segments.
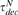	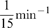	50%–200%	Rate of decay of trophic factor within an axon segment.
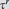	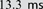 ms	see Results	Time constant governing trophic factor release.
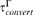	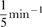	50%–200%	Rate of delivery of trophic factor (synapse resources) to connected synapses.
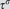	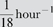	50%–200%	Time constant of neural growth (rate of approach to maximum size)
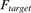	0.2 Hz	50%–200%	Firing rate for collicular neurons (baseline value set approx. equal to RGC firing rates)
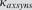	40	50%–200%	Reference number of axon synapses
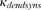	40	50%–200%	Reference number of dendrite synapses
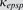	0.5	50%–200%	EPSP produced in immature soma from single, non-potentiated synapse.
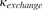	50.0	50%–200%	Maximum exchange rate from trophic factor to synapse resources
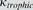	1.0	50%–200%	Relative importance of trophic factor to chemoaffinity in calculating an axon segment's affinity for growth.
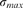	6.0	50%–200%	Maximum growth of neuron, relative to immature size.
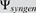	5.0	50%–200%	Amount of axon resource necessary to reach 50% relative synapse generation probability.
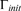	250	50%–200%	Initial level of synapse resources in new synapse.
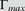	500	50%–200%	Maximum level of synapse resources in a synapse.
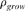	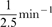	*	Base probability of axon segment growth.
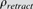	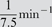	*	Base probability of axon segment retraction.
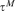	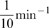	*	Time constant for decay of an axon segment's affinity for growth.
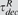	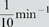	*	Rate of decay of axon resources within an axon segment.
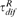	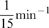	*	Rate of diffusion of axon resources between connected axon segments.
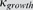	0.2	*	Maximum orthogonal component of axon growth.
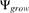	5.0	*	Amount of axon resource necessary to reach 50% relative axon growth probability.
-	13 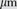	-	Length of axon segment
-	25 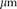	-	Collicular dendrite radius
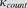	5	-	Maximum number of synapses per axon segment.
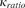	15%	-	Maximum input to a collicular neuron from any single RGC.
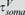	30 ms	-	Time constant of postsynaptic neuron
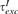	3 ms	-	Time constant of synaptic excitation

Model parameters, their values, and the ranges these parameters were varied over. Values marked by * govern axon growth. Axon model parameters were varied over similar ranges to the other parameters but systematic exploration was not performed. Such changes did not affect the qualitative behavior of the model, but due to insufficient homeostatic balancing, changing of one parameter sometimes required similar changes to another to generate similar patterns of behavior. Parameters governing STDP behaviors were based on data from published studies and were not altered in the present study. Values for STDP parameters are provided in the text. Parameters for the retinal wave model were not systematically explored in this study. The values used to produce waves with specific spatiotemporal properties are provided in Table 3.

### Patterns of retinal activity

**Figure 6:**
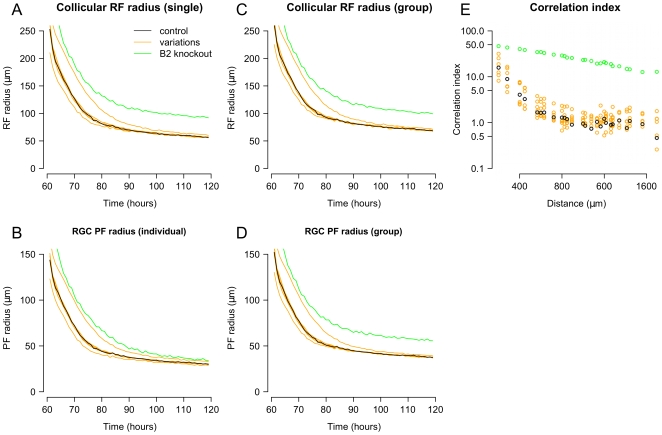
Role of retinal activity correlations on refinement. *A*–*D*. Average RF and PF was measured after changing the patterns of retinal activity. Normal (black) patterns of refinement are plotted against refinement as observed when varying retinal wave properties (orange) and simulated 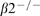 retinal activity (green). Retinal wave properties were varied by increasing and decreasing wave size, velocity and frequency, and this resulted in minimal changes to observed refinement, while simulated 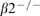 retinal activity patterns produced marked changes to refinement. Receptive field and projection radius resulting from non-correlated retinal activity are not displayed as they did not refine and are beyond the visible regions of the plots (group PF 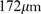; group RF 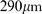). *E*. Measure of correlated activity between RGCs as a function of distance between the cells, plotted on log scale. Changes to retinal wave properties did not significantly affect correlation.

The relative contribution of the different physiological behaviors were analyzed by selectively altering or disabling them. First analyzed was the the effect of changing the spatiotemporal characteristics of retinal waves. The baseline (control) spatiotemporal properties of retinal waves were based on those described for young ferrets [28],[29], as retinal wave properties are similar between species (see [30]) and the size, velocity, frequency and RGC firing properties in ferret are well described. To assess the importance of specific spatiotemporal wave properties to retinotopic development and to see how the selection of control values biased the results, the model was run using patterns of retinal wave activity where the size, velocity or frequency of waves was altered. In all cases, the retinal projection and collicular receptive fields were quantitatively and qualitatively similar (Fig. 6*A–D*; compare orange to black). Baseline waves had a velocity of 180 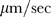, average size of 0.161 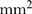, and average interwave interval of 94.2 sec. Ranging the velocity from 112 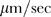 to 466 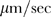, while holding other wave properties largely constant, had minimal effect on retinotopic refinement. Similarly, refinement appeared normal for waves having small (0.101 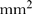) and large (0.428 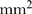) average sizes. Increased wave frequency, as measured by decreasing the interwave interval to 45.1 sec, had minimal effects on refinement. Decreasing wave frequency (interwave interval 202 sec) slowed the rate of refinement but did not have a significant effect on the refined projection.

Mice lacking the 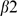 subunit of the acetylcholine receptor have been reported to have significantly altered retinal activity patterns [26],[31],[32] as well as altered retinocollicular projections, with the projective and receptive fields of groups of nearby neurons larger than observed in wild type [33],[34]. In wild type mice nearby RGCs having stronger correlations in activity than RGCs farther apart, while in 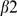 knockout mice (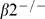) retinal activity is either uncorrelated [31] or strongly correlated over long distances, with RGCs from over a large area of the retina bursting almost simultaneously [26],[32]. In either case, the spatial information provided to refining axons is disrupted, as activity in axons from neighboring RGCs is no longer significantly more correlated than in axons from RGCs located farther apart. To explore the result of this change to retinal activity, simulated 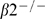 retinal activity patterns were approximated based on data reported by [26] (Fig. 6*E*; green). Using these patterns of RGC activity, the individual RF radius increased by 65% (control: 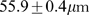 (n = 6 simulations) compared to 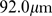 (n = 1 simulation); all subsequent comparisons are reported in this format) and the group RF radius was similarly increased (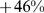; from 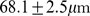 to 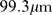). The refined arbor of each individual RGC showed a minor increase in size compared to control (+14%; from 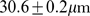 to 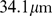), but the group PF radius was increased significantly (+49%; from 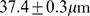 to 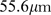)(Fig. 6; *A–D*). These changes are qualitatively consistent with experimental findings [33],[34], but experimentally observed changes are quantitatively much different than observed here (a 2–2.5 fold increase in RF or PF area in the model compared to a 10-fold increase observed experimentally). Several factors might account for this difference. One factor is that, despite strongly enhanced activity correlations between distant neurons, simulated 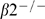 activity still had higher correlations for nearby neurons than for distant ones compared to experimental data [26]. Another factor is that the firing properties of 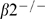 retinas are not fully understood (compare [31],[33] and [26]) and it seems unlikely that 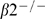 activity is accurately represented here. Simulations in which all RGC firing was decorrelated prevented refinement, suggesting that actual 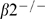 activity is unlikely to be fully decorrelated (e.g., [31],[33]) and there likely exists some distance-dependent pattern of correlation, as indicated by [26],[32]. More generally, the results suggest that it is the correlation patterns between RGCs that drives refinement, not the particular characteristics of retinal waves, and that even small amounts of heightened correlation among nearby neurons can result in nearly normal patterns of refinement.

### Molecular guidance

**Figure 7:**
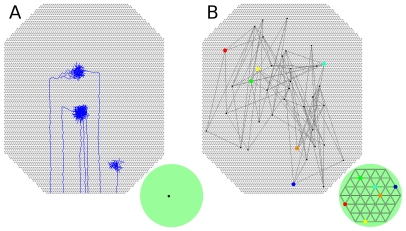
RGC axon development after blocking molecular guidance cues. *A*. Axons from adjacent RGCs project to different collicular areas. Individual axon arbors refine, and neighboring RGCs tend to project to neighboring collicular neurons, but there is no continuity in the axonal projections. *B*. Map of the mature retinal projection (compare to Fig. 1*F*). Six RGCs are marked with different colors, and the center of their projective fields are shown in the colliculus. Global order is completely disrupted. Results from full-sized model are shown.

Molecular guidance cues were implemented as providing axons a bias to preferentially grow near the retinotopically correct area of the colliculus. Disabling this form of guidance by eliminating molecular guidance cues in both colliculus and retinal axons completely disrupted retinotopic organization (Fig. 7). Individual axons did refine, and nearby RGCs often projected to similar collicular areas and had overlapping arbors, but there was no global order in the projections. Disabling chemoaffinity after coarse retinotopic organization was established (i.e. at 60 hours), and allowing subsequent refinement to be driven exclusively by activity-dependent mechanisms, improved refinement (group RF −19% from 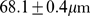 to 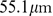; group PF −22% from 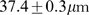 to 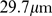). The reason for this improvement appeared to be that while the coarse guidance provided by molecular guidance cues was necessary to guide axons to near their retinotopically correct termination zones, once the axons had arrived, coarse guidance worked against activity-dependent refinement by broadening the area of the arbor where growth occurred. Activity-dependent mechanisms focused axon and synapse growth to the vicinity of synapses that induced spikes in the postsynaptic neuron. Molecular guidance cues worked to increase axon and synapse growth in a relatively broad region of heightened chemoaffinity compatibility, thus diffusing the focusing effect of activity-dependent refinement. These results suggest that molecular guidance cues are critical for establishing initial retinotopic order, but after this order is established, they are not necessary to refine the connection, consistent with an analysis of experimental results [35],[36]. Moreover, it appears that the influence of molecular guidance cues might actually inhibit refinement after axons arborize in the vicinity of their termination zones.

### Spike-timing dependent plasticity

To examine the contribution of STDP to refinement, synaptic plasticity was disabled and all synapses maintained a unitary strength. Unexpectedly, this did not significantly affect model behavior (group RF +0.5% from 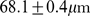 to 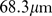; group PF +0.5% from 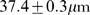 to 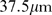). An analysis of the synapses in the unaltered model showed a narrow distribution of synapse weights (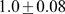; n = 301,343 synapses), so locking synapse weights to unity had little quantitative significance. It is possible that a different implementation of STDP than used here could have more strongly contributed to development, but these results show that STDP, or any form of plasticity regulating the weight of individual synapses, is not required for retinotopic organization or refinement. Plasticity was instead realized through the growth and retraction of individual synapses. LTP and LTD are associated with increases and decreases in the number of synapses, respectively, consistent with the results observed here (see [37]).

Activity-dependent release of brain-derived neurotrophic factor (BDNF) has been linked to long-term potentiation and STDP [38]–[40]. In the model, trophic factor release was linked to STDP, such that trophic factor was released by the postsynaptic terminal proportional to STDP potentiation under a simple pair-based STDP protocol (e.g., [20]). In light of these findings about STDP, the importance of this linkage was investigated by decoupling them and varying the time window for activity-dependent trophic factor release. Specifically, the time window for trophic factor release (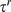 in Eq. 10) was increased by 2, 4 and 8 times (from 13.3 ms up to 106 ms) and the magnitude of trophic factor release was proportionally reduced to account for the longer release window. Trophic factor release was also varied by using a square-wave function, such that if a postsynaptic spike followed within 25 ms of a presynaptic spike, a fixed amount of trophic factor was released (0.532 units, a magnitude that made equal the integrals of square wave and exponential release). In all cases, development was quantitatively and qualitatively similar (maximum changes of +14% PF, +13% RF were observed using the longest time window). Eliminating the activity-dependent mechanism underlying trophic factor release and having trophic factor released on every postsynaptic spike completely prevented refinement, with too much release resulting in very little synapse turnover, as most synapses became stabilized by the trophic factor received, and too little release preventing synapse stabilization and causing very high rates of turnover. These results indicate that activity-dependent trophic factor release, or an equivalent mechanism providing performance feedback to individual synapses, guides the removal of inappropriately targeted synapses and refines the retinotopic projection. The time window for this mechanism, here described as trophic factor release, is consistent with the STDP potentiation window, but is appears not to be restricted to that interval.

### Early appearance of activity-dependent instructive cues disrupts organization

**Figure 8:**
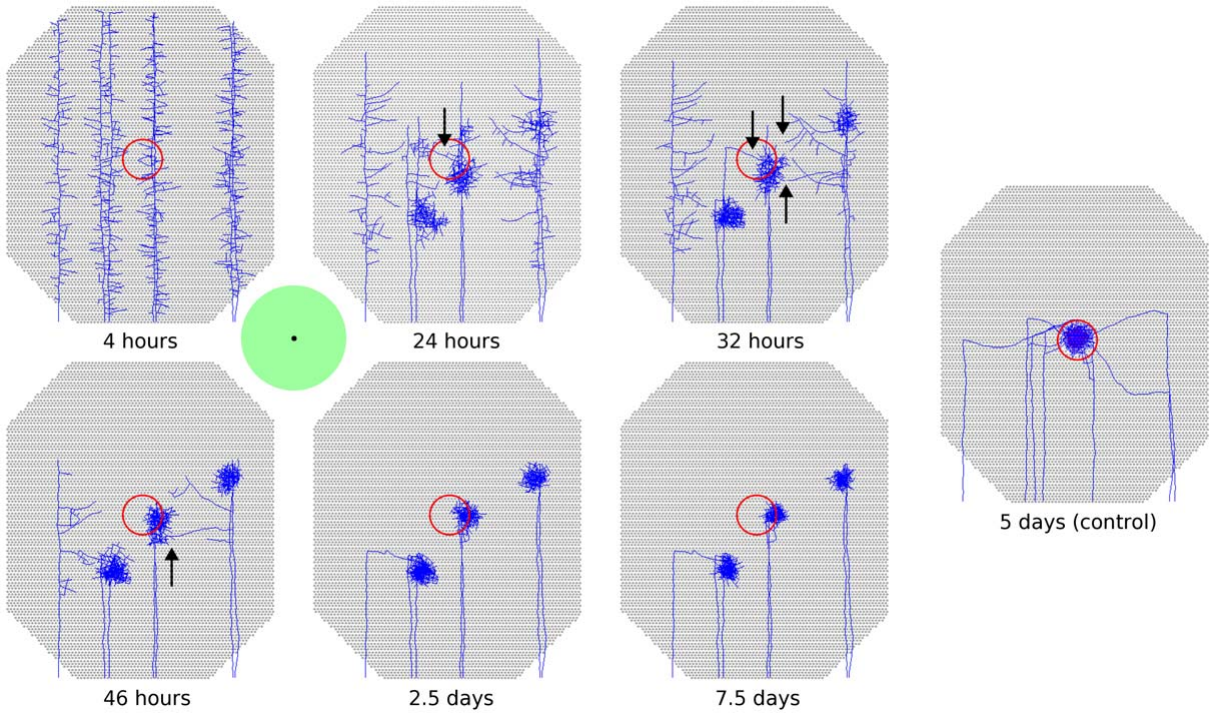
Early onset of activity-dependent mechanisms interferes with retinotopic order. A representative example of development when activity-dependent mechanisms are allowed to mediate axon growth from the time when axons first innervate the colliculus. Axons from seven adjacent RGCs at the retinal center are shown. Red circle indicate retinotopically correct termination zone. Axons begin to refine before they reach the areas of their retinotopically correct termination zones. Despite some axons having branches that extend to near their retinotopically correct location (black arrows), these axon branches are often at a competitive disadvantage for receiving trophic feedback relative to parts of the axon that are in the refining ectopic projections, resulting in retraction of these branches. The location of refinement is strongly influenced by the location of initial axon growth into the colliculus. Once axon arbors refine and innervate a specific location in the colliculus, the location of the arbors appears stable. A control simulation is shown at right. Results from full-sized model are shown.

Development in the model was split into two distinct stages, with axon growth first being mediated by molecular guidance and later, after axons had reached the vicinity of their retinotopically correct termination zones, activity-based feedback began to contribute to guide axon growth. As shown in Fig. 1, this behavior allows nearby RGCs to project to the same areas of the colliculus and to form a refined retinotopic map. While this temporal segregation of roles worked well and is in line with experimental literature [35],[36], initial assumptions of the model were that molecular guidance and activity-based mechanisms both provided instructive guidance from the time when axons first innervated the colliculus. This coincident onset of guidance cues performed poorly, as axon arbors began to refine before they reached their retinotopically correct areas of the colliculus, resulting in numerous ectopic projections (Fig. 8). Delaying activity-dependent instructive cues until after molecular mechanisms had guided axons to the vicinity of their correct termination zones greatly reduced the incidence of ectopic projections and allowed normal organization and refinement to occur. Ectopic projections were sometimes still observed, but these were largely restricted to areas of the collicular boundary. The predominant view in the experimental literature suggests that molecular guidance is required to initially drive axon development, whereafter activity-dependent mechanisms guide refinement [35],[36]. Our findings go beyond this and suggest that, at least during initial development, a separation of mechanisms is necessary.

The effect of early activation of activity-mediated guidance is that it reduces the relative strength of chemoaffinity-mediated growth, as molecular guidance cues become forced to compete with activity-dependent ones. Experimentally, weakening the molecular guidance mechanisms by blocking production of guidance molecules also results in increased ectopic projections (e.g. [1],[2]), consistent with the behavior observed here. Malformed retinotopic projections resulting from early onset of activity-dependent mechanisms were prevented by sufficiently increasing the relative strength of molecularly-driven guidance, allowing both guidance mechanisms to act simultaneously. However, such increases also resulted in accurate axon targeting in the complete absence of activity. This behavior suggests that small animals such as zebrafish, that do not require neural activity for axons to project to their retinotopically correct targets [41] (but see [42]), and whose tecta are a small percentage (2%) of the length of tecta in larger animals, such as chick [24],[43], may not be adversely affected by early onset of activity-dependent guidance. Larger animals, whose tecta (superior colliculi) are much larger and presumably have much shallower molecular gradients, will be impacted more significantly.

### Inter-synapse dynamics

Model synapses required trophic feedback for survival. It was assumed that this mechanism was cooperative, such that trophic factor received by one synapse would also help stabilize nearby synapses on the axon. The theoretical value to such a mechanism, in addition to helping to concentrate synapses to particular areas of an axon arbor, is that trophic feedback from one type of target neuron can help stabilize axonal synapses to different types of nearby neurons (e.g., GABAergic interneurons in retinogeniculate projections), thereby providing a mechanism to spatially align the projection to two (or more) disparate types of target neuron. Functionally similar polysynaptic mechanisms have been hypothesized, such as resulting from rapidly diffusible molecules (e.g., nitric oxide [44]). To evaluate the importance of this assumed behavior, development was examined with the stabilizing effect of trophic factor restricted to the synapse where it was received. Trophic factor receipt continued to influence axon growth and the distribution of axon resources normally. This modification did slow retinotopic refinement, but it also improved the degree of refinement realized (Fig. 4*E*), reducing RF size by 16% (from 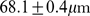 to 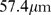) and reducing PF by 13% (from 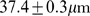 to 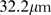). While the ability of synapses to help stabilize their neighbors can affect the rate of retinotopic refinement, it is not required to achieve a refined retinotopic projection.

### Neural representation and neural growth

In addition to the simple integrate and fire model used to represent collicular neurons in this study, previous versions of this model used non-linear integrate and fire neurons (i.e., [45]) and two-compartment neural models, and these changes did not qualitatively affect model behavior (data not shown). To investigate the possibility that neural growth had an influence on retinotopic refinement, collicular neurons were allowed to grow during development, with growth defined as an increase in the resting conductance of the neuron with time, such as occurs with increased surface area of the neuron and dendrite. The effect of such growth was that individual synapses had larger somatic excitatory post-synaptic potentials (EPSPs) on immature neurons than on mature ones. Collicular neuron growth was found to influence refinement in a non-linear way, with maximal refinement observed in neurons having moderate growth (−11% RF and −11% PF compared to control). Further increasing maximal growth reduced refinement. Preventing neural growth, such that the somatic EPSP of neurons resulting from a single presynaptic vesicle release was identical in immature and mature neurons, reduced refinement (+16% RF and +20% PF). It thus appears that neural growth, as exhibited by the decreasing somatic EPSP of individual synapses with time, has an influence on retinotopic refinement. Despite this influence, refinement still appears to be a tolerant process and was observed across a wide range of growth values, and more generally, that retinotopic development remains largely normal despite changes to the mechanisms underlying organization, so long certain core behaviors remain, which are spatiotemporally correlated retinal activity, molecular guidance cues, and activity-dependent trophic factor release.

## Discussion

This study has demonstrated how spiking activity, molecular guidance cues and activity-dependent trophic factor release can guide growth and retraction of individual axons, axon branches and synapses to produce the emergent property of retinotopic organization. While there are many components to the model, its functional behavior is relatively simple. The chemoaffinity-mediated bias for each axon to grow to the vicinity of its retinotopically correct termination zone (TZ) results in axons from nearby RGCs producing diffuse axonal projections near the TZ. The relative density of these axons is higher near the TZ, as is the relative density of the synapses on these axons, even if the synapses from any particular arbor are not well targeted (Fig. 2). Retinal waves cause nearby RGCs to fire together. Because the relative density of synapses from neighboring RGCs is higher near their TZ, collicular neurons near the TZ are more responsive to the activity of these RGCs than are collicular neurons farther away, and hence release relatively more trophic factor to their innervating synapses, stabilizing these synapses. The relative sparseness of synapses on collicular neurons further from the TZ results in them being less able to induce spikes, thus receiving less trophic feedback and retracting. Selective stabilization, along with trophic feedback enhancing synapse and axon growth in the area it is received, produces a self-reinforcing mechanism that results in refined axonal projections. Importantly, it provides a mechanism that enables neural components to use locally available information to generate global order.

The modeling approach used here rests on the assumption that neural development is a process involving several interacting mechanisms and it differs from existing neural development models in many ways, most notably by the degree that it is functionally constrained by biological behaviors not explicitly represented in network-level models (e.g., [14]–[22]), including the physical limitations governing axon growth, the functional requirements of forming and retracting synapses, the spike-based communication employed by neurons, and phenomenological approximations of many physiological behaviors. These constraints allow the contribution of, and interactions between, the different phenomena to be evaluated. It is difficult to examine the role and contribution of these underlying phenomena in models based on abstract descriptions that are open to multiple interpretations (e.g., energy functions) or that only represent one or a few phenomena. For example, if a model representing chemoaffinity and not retinal activity can produce a refined retinotopic map (e.g., [15],[21]), but experiments show that retinal activity is required [35],[36],[46], it follows that either the modeled chemoaffinity representation is functionally incorrect as the model contradicts experimental data, or that the modeled chemoaffinity representation implicitly includes activity and so does not accurately represent molecule-driven guidance. In either case, it is difficult to realistically evaluate either the role or contribution of chemoaffinity using such a model. In more general terms, it has been argued [47],[48] that for a model to have explanatory status, it must replicate the different causal mechanisms underlying the system being modeled, not only reproduce the output. While abstract models that examine only one or a few underlying mechanisms might be useful at providing conceptual insight into map development, the explanatory status of a model, and the detail of its predictions and conclusions, are limited by both the detail of the model and the relative accuracy with which the underlying mechanisms are represented. A detailed model such as described here, which is constrained through representation of a broad range of the phenomena contributing to retinotopic organization, should be better suited than contemporary modeling approaches for examining the role and interaction of different mechanisms underlying development, and for making predictions about these phenomena.

### Core assumptions of the model

The design of the model was based on an analysis of the physiological mechanisms active during development, and the practical biological requirements of these mechanisms. From a physiological perspective, a neuronal projection is defined by the pattern of synapses that exist between bodies of pre- and postsynaptic neurons. The location of these synapses is constrained by the presence of axons, which in turn are constrained by patterns of growth, branching and retraction, as lateral axon motion is not realistic. The growth and retraction of axons and synapses must be governed by locally available information. Neurotrophins, such as BDNF, are prime candidates for mediating this process. Neurotrophins enhance axon growth and synapse numbers [49], are hypothesized to play a role in synapse stabilization and maintenance [50]–[52] and are released in an activity-dependent manner [39],[51]. The effects of BDNF are local to the area released [53], may be synapse specific [13],[51] and postsynaptic activity within tens of milliseconds of presynaptic activity results in synaptic enlargement in a process mediated by BDNF [54]. Molecular guidance cues also influence axon growth [55] and more generally, cellular behaviors are influenced by variations in firing rates (e.g. [56]). Based on these points, two core assumptions were derived to guide model behavior:

1) Axon growth and branching, and synapse formation, had increased probabilities in areas of an arbor with greater relative (a) chemoaffinity compatibility with surrounding tissue than other sections of the arbor, and (b) trophic feedback to the presynaptic terminal, which was provided by the postsynaptic terminal when a postsynaptic spike followed shortly after a presynaptic spike.

2) Synapses required trophic feedback for survival, and synapses with insufficient trophic support were eliminated.

To implement these principles, additional considerations were required, such as how to regulate the size of the axon and the synapse population. This led to three further assumptions:

3) To limit total axon arbor size, axons required a regulated substance, referred to here as axon resources, in order to grow and to persist. Axon resources were produced in finite quantities by the soma and were delivered preferentially to regions of the arbor with higher relative chemoaffinity and to near synapses receiving relatively more trophic feedback. A reduced presence of axon resources resulted in an increased likelihood of axon retraction.

4) To control the number of axonal synapses, the probability of new synapse formation was decreased with increased numbers of existing synapses, and each synapse required increasing amounts of trophic factor to survive with increasing numbers of synapses on the axon.

5) The number of dendritic synapses was controlled through direct and indirect means. The more synapses present on a dendrite, the less likely the dendrite was to accept new synapses. When a collicular neuron's average firing rate (integrated over tens of minutes) was above its target level, it both became less likely to accept new innervating synapses, and existing synapses decreased the trophic feedback provided to presynaptic terminals in order to induce some innervating synapses to retract.

The model was based on implementation of these mechanisms. The first two assumptions were core to the model's behavior and so it was not possible to carefully evaluate alternatives. The others assumptions were tolerant to variation so long as the behavior that these assumptions were designed to produce was realized (as assessed through both parameter variation and unpublished versions of this model). Homeostatic mechanisms were found to be important in the model design. The complexity of the model made it a practical impossibility to pre-define numerical quantities for the large range of mechanisms represented, such as exact EPSP magnitude, the number of synapses, total axon length, trophic feedback quantities, etc. Even when it was possible to define specific values for a quantity, minor modifications to the model often made such selections inappropriate, forcing the parameters to be readjusted. Defining quantities loosely and in such a way that they were subject to dynamic regulation (e.g., assumptions 3–5) produced a system that was very tolerant to perturbation. The same issues encountered in producing this model are also observed by nature, as there is a high degree of variability that can arise from genetic and environmental factors, and the biological system is tolerant to perturbation and it preserves its functionality despite changes to the mechanisms underlying this functionality [57].

### Spike-timing dependent plasticity

The finding that STDP was not required for retinotopic refinement was unexpected. On reflection, this finding is consistent with the results of several experimental studies. Synaptic plasticity saturates after 60–100 spike pairings [7],[58], meaning that synapses that are already maximally potentiated for a given interval between pre- and postsynaptic spikes do not further potentiate. The fact that it is possible to observe significant synaptic potentiation and depression in STDP studies therefore suggests that most synapses exist in largely non-potentiated states, for otherwise such potentiation would not be observable in them. The notion that synapses are not significantly potentiated or depressed in their normal state is reinforced by findings that artificially induced STDP is lost if cells are allowed to resume their normal firing patterns [59] and that the distribution of individual synapse strengths is unimodal [60]. Further, experimental studies have indicated that it is either the timing of bursts between pre- and postsynaptic neurons, or the coincidences of individual spikes, that underlies plasticity, not STDP [61].

**Figure 9:**
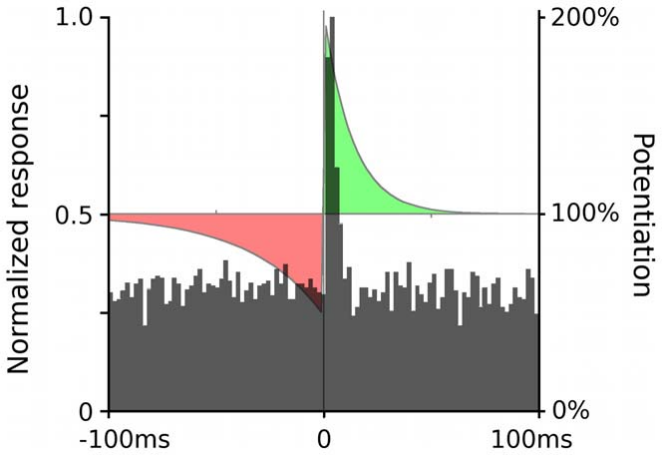
Cross-correlogram and STDP. Background shows the cross-correlogram (CCG) between an RGC and a monosynaptically connected collicular neuron (final 3 hours of development). The spike times of the collicular neuron are shown relative to the firing of the presynaptic RGC. There were 9 synapses between this particular pair of cells, and the innervating RGC represents 8% of the excitatory input to the collicular neuron. The overlaid STDP plot showing synaptic potentiation (green) and depression (red) as a function of the interval between pre- and postsynaptic spikes. The peak of the CCG is aligned with the maximal potentiation response in the STDP plot, indicating a strong potentiating force. However, each of the spike pairs indicated in the CCG is likely to influence the potentiation level of the synapse based on the STDP function, significantly moderating any potentiation realized.

Cross-correlograms (CCGs) between pairs of monosynaptically connected cells often show a number of uncorrelated spike pairs and a peak a few milliseconds offset from time zero (Fig. 9; e.g., [62]), indicating that the postsynaptic cell has a higher than average probability of firing immediately after the presynaptic neuron, a behavior observed in modeled neurons. This CCG pattern should result from any system where there are several innervating neurons for each target neuron and when a spike in the presynaptic neuron is followed by a spike in the postsynaptic neuron only infrequently. While the peak in the CCG between monosynaptically connected cells is typically in the optimal location for inducing maximum potentiation in the synapse, the relatively large number of non-correlated firings would be expected to have a counteracting and depressing effect on plasticity. Further, when observing saturation of plasticity constraints, where maximal plasticity appears to be approached asymptotically [7],[58], the more a synapse is potentiated the less potentiating force there is after every pre-post spike pair. Depressing spike pairs (i.e., post-pre) are far from their saturation level and so are likely to maintain full potency, further inhibiting strong potentiation.

Manipulating the strength of individual synapses is not the only way to vary the effective strength of a monosynaptic connection. As indicated in Fig. 3*C*, variation in the strengths of synaptic coupling between two neurons can be achieved by variable numbers of synapses between the cells. Increasing or decreasing this number alters the effective strength of the “monosynaptic” connection. Based on the interpretation that “synapse” plasticity is realized by altering the number of synapses between neurons, the model's use of trophic factor release to regulate synapse stabilization, retraction and growth is consistent with the single-spike coincidence plasticity rule of Butts et al. [61], if plasticity is considered to represent the formation and retraction of synapses rather than the modification of the weight of individual synapses. The timing of individual spikes in pre- and postsynaptic neurons is important for plasticity, and the plasticity realized is Hebbian in nature, but it appears to not be realized by the changing of the efficacy of individual synapses.

It is possible that the implementation of STDP used here is too restrictive and that a different implementation could more strongly contribute to retinotopic organization and refinement. However, as argued above, STDP may not play a significant role in development. If that is the case, why is it a seemingly ubiquitous phenomenon? The neurotrophin BDNF has been associated with STDP and LTP [38]–[40]. Further, BDNF in the presence of glutamate mediates enlargement of synaptic spines in hippocampal slices [54], while LTP is associated with an increase in the number and size of synaptic spines, and LTD is associated with spine shrinkage and retraction [37]. It is entirely possible that what is observed as STDP experimentally is actually the byproduct of another mechanism, such as synapse stabilization. Using the retinocollicular projection as an example, numerous synapses are created during development but the only ones that persist are those that produce the refined retinotopic projection. There must exist a mechanism to remove inappropriately targeted synapses. One mechanism to accomplish this, as demonstrated here, is the activity-dependent release of trophic factors, where synapses contributing to a spike in the postsynaptic neuron receive trophic support and stabilize, while synapses receiving insufficient trophic factor retract. The timing of trophic factor release in the model is consistent with the time window for STDP potentiation. What is observed experimentally as STDP, at least in retinotectal synapses, might be an experimental artifact of a process relating to synapse stabilization and retraction, with what is observed as potentiation reflecting a mechanism that stabilizes synapses and depression reflecting a mechanism that makes the synapse more likely to retract. It is also possible that STDP is a redundant or complementary to another mechanism, or that it plays a functional role that was not examined in this study (e.g., [63]).

### Axon growth and gradient detection

The extent and direction of axon growth in the model was mediated by probabilistic growth and retraction. Areas of an arbor with higher chemoaffinity compatibility with their surroundings, and/or increased trophic feedback, were more likely to extend and branch, and areas with relatively lower amounts were more likely to retract. During chemoaffinity-mediated growth, this mechanism was sufficient to produce a coarse retinotopic projection (Fig. 1*C, D*). After activity-dependent feedback began to influence axon growth, this same principle was able to generate refined arbors in the retinotopically correct termination zone. What is notable about this finding is that both chemoaffinity and activity-based axon guidance can be mediated by the same functional mechanism, and that the gradient detection and tracking of extracellular molecules by growth cones was not required during arborization and refinement. We note that growth-cone mediated guidance is still required for an axon to reach the colliculus and extend to its posterior pole.

Axon growth cones can detect gradients with remarkable sensitivity [64],[65], however it is not clear that the expression of guidance molecules is sufficiently smooth at the cellular and sub-cellular level to support such accurate guidance during retinotopic organization and refinement, especially considering that similar guidance molecules are expressed not only on collicular neurons but also on innervating axons [24], that measured mRNA levels for guidance molecules may not be locally smooth [1],[66] and that there may be non-uniformities in the density of axons and dendrites. If an axon is able to sense the relative difference in chemoaffinity compatibility in different parts of the arbor, and use this to influence the relative likelihood of local growth in the arbor, the arbor is effectively able to act as a very large gradient detecting growth cone. This behavior has been previously postulated for chick tectal development [43]. Such a mechanism could guide axon growth in the presence of shallower gradients, or in a noisier environment, than possible by gradient detection in individual growth cones.

Axon growth in the model does phenomenologically approximate experimentally observed patterns of axon growth, with initially coarse arbors refining into retinotopically ordered projections in the presence of normal retinal activity patterns (e.g., [33],[49]), and that the number of synapses and axon branches increase with exposure to trophic factor [49]. However, the implementation is very simplified compared to biology. Physiologically, there are interactions between the molecular machinery underlying chemoaffinity and trophic factor influence on axon growth (e.g., [67],[68]) and it is possible that activity-dependent influences are present throughout axon arbor development, and also that trophic factors help regulate the influence of molecular guidance cues [68]. On the other hand, molecular guidance cues could simply be sharing the same signaling pathway as trophic factors, and despite this molecular overlap between mechanisms, both could remain functionally distinct. Similarly, only the positive effects of trophic release were represented, not the opposing behaviors of trophic factors, where mature forms of the molecules promote growth, and the immature uncleaved molecules, such as proBDNF, promote synapse and axon retraction [37]. While a more mechanistically accurate model of axon growth will provide better insight into the molecular interactions involved in signaling axon and synapse growth and retraction, we found nothing to indicate that our phenomenological approximation of axon growth would be significantly different with a more mechanistic representation, nor that a more mechanistic representation would alter our findings on the overall behavior of growing axons.

### Local excitation and distal inhibition

Retinotopic development has been the subject of many computational models [69], and these models have been used to help identify the functional mechanisms necessary for development. In order to produce an ordered projection, the majority of these models (but not all, e.g., [18]) assume lateral connectivity between target neurons, where typically activity in one neuron results in excitation of nearby neurons and inhibition of neurons farther away (see [69]). This excitation/inhibition mechanism imposes architectural requirements on what is necessary for organization, and the high reversal potential for chloride early in development [70] suggests that lateral inhibition is not realistic, as synapses traditionally considered inhibitory (e.g., GABAergic) would be excitatory during the period of retinotopic organization and refinement.

In this study we have found that lateral synaptic connectivity was not required for producing an ordered retinotopic map, simplifying the theoretical functional requirements of the developing network. Simulated axons from neighboring RGCs were able to target the same collicular neurons based on their correlated firing properties and on the stabilizing effects of trophic factor. Synapses from RGCs stabilized on collicular neurons that were responsive to their activity by means of increased trophic factor receipt (Fig. 2*B*). Correlated activity between nearby RGCs resulted in axons from nearby RGCs targeting the similar collicular neurons. Over the course of hours of simulated time, this mechanism results in increased axon and synapse growth in the area where more trophic feedback was received and these new synapses targeted nearby collicular neurons, focusing the axon projection. Collicular neurons sought to maintain a target firing rate, producing a normalizing force that limited the number of synapses present. Because of these factors, the resulting projection was ordered at the global level (Fig. 1*F*) though was subject to scatter at the local level (Fig. 1*G*).

### Conclusions

The focus of this study was to examine the behavior and interaction of the mechanisms underlying neural development, and the approach here follows that used in the modeling of other complex phenomena, most notably climate [71]. Both climate and neural development are examples of complex systems, and predictive and descriptively accurate models of such complex systems may themselves be complex and not necessarily capable of being simplified to a simple or mathematically analyzable form. Climate models represent approximations of many of the causal mechanisms underlying weather, such as radiation, cloud cover, humidity, momentum, sea surface temperature and pressure gradients [71],[72]. The model described here addresses retinotopic organization and refinement as being causally produced from phenomenological approximations of many mechanisms known to be active during development of the retinocollicular projection. Two very important mechanism are the growth, branching and retraction of individual axons, and the durability of individual synapses. Axon growth is a process underlying the formation of all neural projections, and axons have extremely restricted movement once extended through neuropil. Synapses must retract based on information available to each individual synapse. A descriptively accurate model of retinocollicular development requires consideration of the physical constraints posed on development by these and other mechanisms.

The model represents many physiological phenomena active during development in as simple a form as practical while still approximating the functional behaviors of the phenomena. The lack of detailed representation of these mechanisms can be justified, we would argue, because the details of the mechanisms can vary between species though the developmental outcomes are similar. For example, similar patterns of retinal waves are observed in many species yet their statistical and molecular details vary (see [5],[30],[73]). Likewise, chemoaffinity gradients are a common phenomenon but they are mediated by different molecules in different species [24]. Despite these differences, similar patterns of retinotopic organization persist. It stands to reason that it is the commonalities of behavior observed between species that are important for producing the common patterns of development, not what are essentially biological implementational details. The results of this study support such a conclusion, as qualitatively similar development was observed despite variations and perturbations to the model. It was only when key functional mechanisms were disabled that the model failed to produce retinotopic organization or refinement.

Predictions of the model include that:

- the appearance of activity-dependent instructive cues before RGC axons have arborized in the vicinity of their retinotopically correct termination zones disrupts retinotopic organization;
- experimental manipulations that block the plasticity of individual synapses (e.g., STDP) while leaving other mechanisms intact should have little effect on refinement of an ordered retinotopic projection;
- during axon arbor growth and refinement, blocking gradient detection abilities of axon growth cones will have little effect on arbor development or the refinement of an ordered retinotopic projection;
- lateral connectivity between collicular neurons is not functionally required to achieve retinotopic order or refinement;
- altering the spatiotemporal properties of retinal waves will not appreciably affect retinotopic refinement so long as the distance-dependent correlational structure of the activity is preserved;
- activity-dependent release of trophic factor, possibly synapse specific, is required for directing synapse removal and patterns of axon growth, and blocking this mechanism will prevent axon arbor refinement; and
- conductance changes of the postsynaptic neuron as would occur during neuron growth influence retinotopic refinement, and other things being equal, retinal axon development in a mature colliculus will produce a less refined projection than development in a colliculus while collicular neurons are themselves growing.

Although the model is restricted to the retinotectal/retinocollicular system, the phenomena represented in it are found in neurons throughout the brain, and the findings here may apply more broadly. With minor modifications, the model is potentially applicable to the description of development in different brain areas. Explicit representation of many physiological mechanisms allows the model to be more easily compared to and constrained by physiology than most contemporary modeling approaches. It may be that the most predictive and descriptively accurate models of retinocollicular development, and of neural development in general, will resemble the approach described here, incorporating phenomenological approximations of many physiological mechanisms, in particular explicit representation of the growth and retraction of individual axons and synapses.

## Methods

### Overview

**Figure 10:**
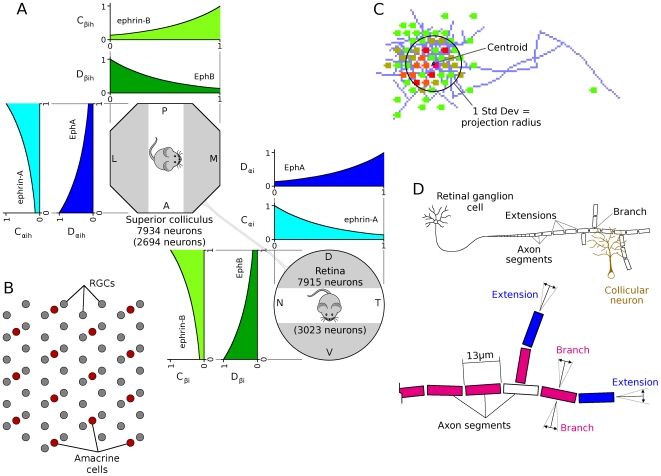
Chemoaffinity, retinal layout and axon growth. *A*. The expressions of ephrin and Eph gradients were approximated by simple exponential functions, 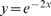 and 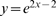. The expression of ephrins was represented by the variable “
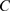
”, with the subscript indicating the family (i.e. 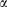 for 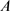 and 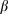 for 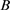), and expression of Eph receptors are represented by the variable “
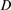
”. *B*. Arrangement of neurons in the model retina. Spontaneous retinal activity was produced by the activity of simulated amacrine cells [30]. An additional layer of retinal ganglion cells (RGCs) was added to the model, with RGC density being four times that of amacrine cells, comparable to RGC/amacrine cell ratios in P6 ferret, after RGC levels become stable [83]. For simplicity, a uniform pattern of cell spacing was assumed. The response of each RGC was estimated based on the wave behavior at the location of the nearest amacrine cell. *C*. The size of an axonal projection was measured by the spatial distribution of neurons that the axon projected to. To do this, the centroid of these neurons was calculated, weighted by the number of synapses projecting to each neuron, and the standard deviation of the distance of each neuron to the centroid was calculated The projection radius was defined as this standard deviation. An equivalent mechanism was used to measure the spatial distribution of RGCs projecting to each collicular neuron, which was defined as the receptive field radius. *D*. Cartoon of axon, showing axon segments, extension and branching. Axon extension occurred at axon tips (i.e., segments that had no children, in blue), and branching occurred in segments that had already extended but that did not have any branches (red). Axon retraction occurred only at axon tips. Extending axons grew largely in-line with the existing axonal trajectory, and branching occurred in a largely orthogonal direction (details in text).

The structure of the model is shown in Fig. 10*A*. A circular retina composed of 7915 RGCs projected to an octagonal colliculus having 7934 neurons. Neurons in both retina and colliculus were distributed on a hexagonal matrix. The model retina was circular (diameter 1.6 mm) and the colliculus had 110 rows of neurons with each row having 80 neurons (0.8 mm×0.94 mm), with the corners of this rectangle truncated. In a reduced size version of the model that was used for model analysis, only the central 30% of the simulated retina and colliculus were modeled (Fig. 10*A*, white rectangular areas). The smaller model had 3023 RGCs projecting to 2694 collicular neurons. The model was not sensitive to small changes in the ratio of retinal to collicular neurons, but this was not systematically explored. Map compression and expansion was examined in a previous version of this model [74]. The dendritic radius for each collicular neuron was 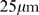. The soma of collicular neurons was considered to reside at the center of the dendritic arbor.

Axons in the model were represented as a connected series of segments, each 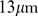 in length, a size selected to be sufficiently small to allow for realistic patterns of growth but large enough to make the model computationally tractable. Each axon segment was able to extend and branch, and retraction occurred at axon tips (Fig. 10*D*). Axon segments were considered to have an “affinity” for their surroundings, which determined their propensity to grow, sprout synapses and retract. Axon segments required resources to grow, and the availability of these resources was managed by the soma. Segments received an amount of growth resource that was a function of the segment's affinity. Axons with higher amounts of growth resources were more likely to extend, branch and generate new synapses, while segments with lesser amounts were more likely to retract. To achieve self-limiting axon growth, each soma was assumed to have a finite amount of growth resources that was distributed throughout the arbor.

Simulations began with each RGC axon extending along the A-P axis of the colliculus, corresponding to development as seen in P1 mouse [33]. Initial axon placement had each RGC axon entering the colliculus at the anterior side and extending along the length of the anterior-posterior (A-P) axis, in a position along the lateral-medial (L-M) axis that corresponded the the RGCs location along the retinal dorsal-ventral (D-V) axis. The exact L-M position varied by a random amount (a Gaussian distribution with mean zero and standard deviation of 20% of the width of the colliculus). This design was based on descriptions of mouse and chick retinocollicular development [24],[34],[43]. The colliculus had flat sides both so axons could linearly project along the collicular boundary and so the model did not rely on an isotropic projection from retina to colliculus. The orientation of axon segments in the initial projection was parallel to the A-P axis except for a small random variation. Specifically, the orientation of each segment was described by 2 vectors, one of unit length and parallel to the A-P axis, and a second perpendicular vector whose magnitude was a uniform random variable selected on the interval [−0.2, 0.2]. Subsequent branching and growth occurred as described below.

Development occurred in two stages, each lasting 60 hours of simulated time. During the first 60 hours, development was mediated by chemoaffinity, and interstitial branching and subsequent growth was guided only by the differential in chemoaffinity compatibility across the arbor. During the second 60 hours, trophic feedback and chemoaffinity both contributed to growth. While synapses may be present throughout axon development in the colliculus, synapse creation in the model was inhibited until the onset of trophic feedback influence on axon behavior (i.e., 60 hours development time) as synapses had no influence on axon growth before this time. This allowed the first 60 hours of axon growth to be pre-computed and used as a starting point for simulations of the second development stage, reducing the computational requirements of the model. Quantitative analysis as reported in Results was performed at 119 hours as synapse generation was turned off during final hour of the simulation to assess the stability of synaptic projections and to passively allow poorly targeted synapses to retract (e.g., note removal of mistargeted synapses at 120 hours in Fig. 3*C*). PF and RF sizes were reduced as a result of passive pruning, but the projections were qualitatively similar (data not shown).

The excitation level of model neurons were updated on every simulation clock cycle (1 ms) while synapses were updated only on the occurrence of a pre- or postsynaptic spike. When a neuron fired, it cycled through all its axonal synapses, “pushing” excitation onto the target cell of each, and updating synaptic potentiation based on STDP learning rules for a presynaptic spike. The neuron then cycled through its dendritic synapses, updating synaptic potentiation based on the occurrence of a postsynaptic spike. To improve simulation performance, many cellular behaviors were updated less frequently. Equations relating to axon growth, branching and retraction, and to synapse growth were recalculated every 5 sec simulated time. Equations relating to synapse resources, synapse retraction, axon resources, homeostatic controls and intra-axon diffusion were recalculated every 0.5 sec. With the exception of millisecond calculations (e.g., EPSP summation, STDP and trophic factor release), the model was not dependent on the interval between updates. Different intervals were used in some simulations and no change to model behavior was observed.

Previous versions of this model ([74] and unpublished) used mathematically different but functionally similar representations of these mechanisms and produced qualitatively similar results.

#### Mathematical conventions

An element in an equation that has a lower and/or upper bound was indicated by square brackets with a trailing superscript and/or subscript to indicate the bound. For example, 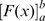 is bounded on [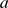, 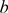], meaning that the value of this term in an equation cannot fall below 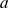 or rise above 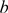. Similarly, 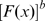 has an upper bound of 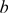 and no lower bound, and 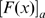 has a lower bound of 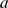. The number of elements in each set was indicated by use of the absolute value symbol, such that 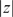 was the number of elements in set 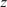. Some variables used both super and subscripts to indicate their function. Lists of the variables and parameters used by the model are in Tables 1 and 2.

**Table 2:** Variables and functions used by the model.

Variable	Defined	Description
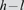	-	Subscripts for presynaptic neuron (i), postsynaptic neurons (j), axon segments (h,l) and synapses (k).
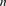 , 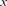	-	General variables.
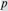	-	Probability.
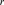	Eq. 10	Trophic factor received by the presynaptic terminal.
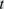	-	General time variable.
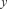 , 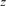	-	Sets of synapses ( 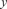 ) or axon segments ( 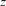 ).
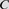	Fig. 10	Ephrin gradient.
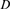	Fig. 10	Eph gradient.
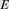	Eq. 1	Sigmoid-like function 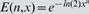 .
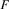	-	Firing rate of neuron.
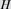	Eq. 14	Homeostatic scaling factor of excitatory input.
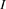	Eq. 18	Total synaptic input to a neuron.
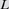	-	Simulated calcium imaging response from retinal wave activity (from [30]).
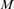	Eq. 2	“Affinity” of axon segment, indicating the affinity of an axon segment to its surroundings.
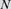	Eq. 11	Trophic factor present in axon segment.
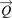	-	Orientation of existing axon segment.
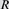	Eq. 3	Axon resources present in segment.
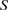	Eq. 21	Saturating level of STDP potentation/depression.
V	Eq. 17	Excitation level of neuron (resting value = 0).
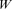	Eq. 20	Excitatory strength of synapse.
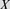	Eq. 15	Trophic factor to synapse resource exchange rate.
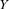	Eqs. 12–13	Chemoaffinity score of axon segment.
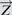	Eq. 6	Direction of growth of new axon segment.
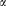 , 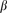	-	Subscripts indicating ephrin/Eph gradients.
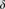	-	Number of action potentials in neuron over previous 500 ms.
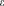	Eq. 19	STDP efficacy (from [8]).
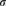	Eq. 16	Conductance of neuron, dendrites and synapses. Total resting conductance of immature neuron = 1.0.
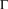	Eq. 9	Level of synapse resources present in synapse.
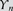	-	Normal random number (Gaussian distribution) with mean of 1.0 and standard deviation 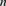 .

Variables and functions used by the model, including where they are defined (where applicable) and a brief description.

Several formulas in the model utilize a sigmoid-like function that has a stable, near-unity value for small 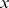 and that decays to zero with increasing 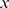. The following family of functions was used for these cases:
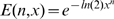
(1)This function has the value 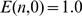 and 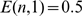 for all positive 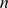. The flatness of 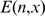 for low 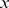, and the steepness of its decay, varies with 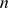. 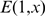 is standard exponential decay with a half-life of 1.0. The symbol 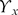 represents a random number with a Gaussian distribution of mean 1.0 and standard deviation of 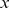.

#### Analysis of retinotopic projection

The radius of each RGC's projective field (PF) is defined as the standard deviation of the distance of collicular neurons that the RGC projects to from the centroid of these neurons (Fig. 10*C*). The size of each collicular receptive field (RF) is calculated similarly, based on the location of innervating RGCs. The reported PF and RF sizes were the average of all RGCs or collicular neurons, respectively. To examine the continuity at the local level, a separate measurement for PF and RF size was made by measuring the collective PF and RF for groups of 19 neighboring cells from all locations in the retina and colliculus that was two or more cells distant from the border. This was 7279 and 2503 RGCs for full and reduced size models, respectively, and 7278 and 2173 collicular neurons in full and reduced size models, respectively. To analyze global mapping performance, a “normal” projection was generated by averaging the PF location of each RGC over five control simulation runs. The global mapping performance of subsequent simulations was measured by averaging the distance of each RGC from its normal location. All results in the text are reported as 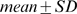 unless otherwise indicated.

#### Implementation and runtime

The model described here was implemented in multi-threaded C++. Simulation data was saved in an embedded database (sqlite). Data analysis was performed on the database, and simulations could be resumed based on data stored there. All simulations were run on desktop computers (Intel Core 2 duo and quad; Intel Pentium D duo) running a 64-bit debian based operating system (debian lenny and Ubuntu). Simulation runtimes took 1–6 days, depending on simulation size and hardware used. The full-sized model required 5–6 days on a quad-core CPU, and the reduced size model 1–2 days on a dual core CPU.

### Model components

#### Axon model

The model of axon growth described here was designed to phenomenologically approximate retinocollicular axon arbor development in as simple a mechanism as found possible. Different axon models that had similar functional behaviors (i.e. axon growth and branching being more likely in parts of the arbor having increased trophic factor receipt and/or enhanced chemoaffinity compatibility) but having different algorithmic implementations achieved qualitatively similar results (e.g., [74] and unpublished). Axons were composed of a connected series of segments, each 13 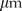 in length. Each axon segment could support up to two child segments. Axon growth occurred through the creation of child segments and retraction occurred through removing axon segments. The affinity, 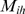, of axon segment 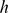 in neuron 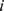, was:
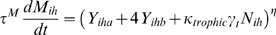
(2)where 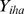 and 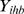 were the relative chemoaffinity scores for ephrin-A/EphA and ephrin-B/EphB gradients, respectively (Eqs. 12 and 13), 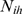 was the trophic factor present in 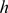 (Eq. 11), 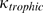 was a scaling constant to regulate the relative importance of trophic factor in calculating affinity, and 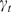 represented the time-dependent sensitivity to trophic factor. Unless otherwise noted, 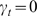 until trophic feedback began to influence axon growth (i.e., at 60 hours) and then linearly increased to 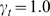 by the end of simulated development (i.e., 120 hours). The time constant, 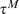, was relatively long (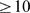 min) to average out short term fluctuations in trophic feedback. To better phenomenologically reproduce axon behaviors, in particular to limit excessive interstitial branching and to allow axons to better extend to their correct location on the L-M axis, the affinity of axon segments with two children was reduced by 20%. The multiplicative constant 4 on 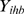 was used for a similar purpose. The non-linear scaling term 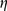 was 1.5 as this most accurately generated reported patterns of axon growth [24],[33]. Values from 1.0 to 2.0+ were also viable, with higher values producing increasingly refined arborizations both under chemoaffinity driven growth and under activity-dependent refinement.

The resources necessary for axon growth (e.g. molecular, metabolic, etc.) were distributed to axon segments proportional to their affinity score, resulting in axon segments having a higher affinity receiving proportionally more growth resources. Axon resources, 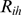, in axon segment 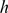 of neuron 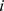, accumulated based on the segment's affinity, resource diffusion between neighboring segments, and decay:
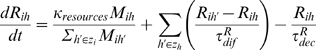
(3)where 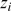 was the set of axon segments from neuron 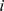, 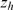 was the set of axon segments connected to 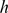, and 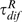 and 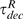 were diffusion and decay constants, respectively. Eqs. 2 and 3 were updated every 500 ms. The value 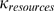 was the total amount of axon growth resources distributed throughout the arbor every update step, and it had a direct relationship to total arbor size. The magnitude of 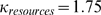 was selected to produce realistic patterns of arbor growth. There were no homeostatic factors governing axon resource distribution and the model was sensitive to changes of this parameter. Appropriate values were selected based on axon segment length and collicular size, and a reasonable approximation was 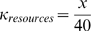, where 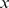 is the number of axon segments required to stretch across the A-P axis of the colliculus.

Axon growth and retraction were functions of the amount of axon resources present in a segment. Growth occurred probabilistically when resource levels were above unity, and retracted probabilistically when below unity. Specifically, the probability of growth, 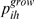, for axon segment 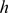 of neuron 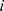, was:
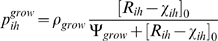
(4)where 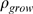 was the peak probability of axon growth, 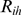 was the amount of axon resources present in segment 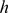, 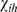 was the growth threshold (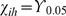 for growth and 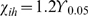 for branching), and 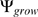 was a constant controlling the sensitivity to axon resources. The threshold for axon branching was 20% higher than for extension under the assumption that growth in a segment was more likely to occur than branching. Axon retraction only occurred in axon segments that had no child segments (i.e. axon tips). The probability of axon retraction, 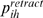, was:
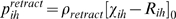
(5)where 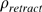 was the peak probability of axon retraction. Eqs. 4 and 5 were calculated every 5 sec.

The direction of axon growth varied for extension and branching. Axon growth occurred in axon segments with no children, and the direction of growth was similar to the existing trajectory of the axon. Growth through interstitial branching occurred in a largely orthogonal direction, and growth through branching only occurred in segments having one child segment (Fig. 10*D*). The vector indicating the orientation of segment 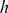 was 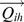. The vector 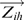 indicated the direction of growth for the new segment:
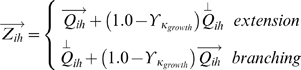
(6)where 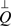 was a vector perpendicular to 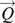 and 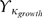 was a normal random variable with mean of 1.0 and standard deviation 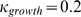. Axon segments with two children had no further growth until one of the child segments retracted. A newly formed segment had no axon resources. When a segment retracted, its resources (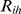) were absorbed into its parent segment. When a segment extended or branched, its resource level was decremented by 1.0.

#### Synapse model

Synapses were formed by co-operative activity of both axon and dendrite. Synapse survival was mediated through synaptotrophins [50]. Each presynaptic terminal received trophic factor when a spike in the postsynaptic cell followed vesicle release in the synapse within tens of milliseconds. To implement synapse survival in a way that was homeostatically regulated, each presynaptic terminal was considered to require resources from the soma (e.g., molecular, metabolic, etc.) in order for the synapse to survive. Each synapse started with an initial resource allocation, expended resources on each presynaptic spike, and retracted when its supply of resources was exhausted. Resources were replenished in the presynaptic terminal through conversion of received trophic factor into synapse resources. The exchange rate of trophic factor for synaptic resources was regulated by the soma.

The resource-based mechanism implemented here for synapse survival is much simpler than occurs in nature, where the mature form of trophic factors (e.g., BDNF) may promote synapse survival while the immature forms (e.g., proBDNF) may induce retraction [37]. Biologically, it seems plausible that instead of synapses being weakened with each vesicle released, they are instead weakened by proBDNF that is released on synapse activation, and reinforced when there is coincident postsynaptic activity. As this behavior is functionally equivalent to the described resource-based mechanism, the simpler mechanism was implemented.

The probability of synapse formation, 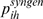, in axon segment 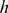 of neuron 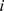, was:
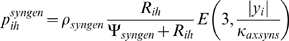
(7)where 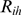 was the axon resources present in segment 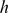 of neuron 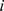 (Eq. 3), 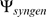 was a constant controlling the sensitivity to axon resources, 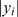 was the number of axonal synapses in neuron 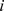, and 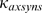 was a soft target of the number of synapses on the axonal arbor. The function E( ) was a sigmoid-like function (Eq. 1). Synapse growth occurred only in synapses having less than 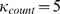 synapses on the segment to prevent an unrealistic number of synapses being created per segment.

Every 5 seconds, each axon segment had a probability, 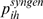, of attempting to generate a synapse with a local dendrite. When this happened, a dendrite was selected at random from the set of dendrites overlapping the axon segment and this dendrite was queried to see if it would accept a synapse from this particular RGC. The probability, 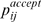, that the dendrite of postsynaptic neuron 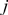 would accept a new synapse from presynaptic neuron 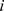 was:
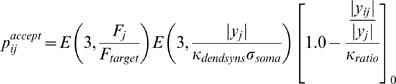
(8)where 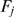 was the firing rate of collicular neuron 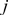, 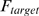 was the target firing rate, 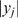 was the number of synapses on the dendrite of neuron 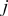, 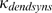 was a reference number of synapses, 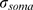 was the size (as measured through relative conductance) of the postsynaptic soma and dendrite (Eq. 16), 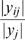 was the ratio of dendritic synapses on neuron 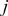 that are from RGC 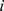 and 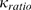 was the maximum ratio of dendritic synapses that could originate from a given presynaptic neuron. A small value of 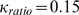 was used in all simulations here to force each collicular neuron to be innervated by several RGCs. Larger values for 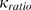 produced increasingly refined retinotopic projections (unpublished observations), with 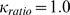 producing much more refined retinotopic projections than that described in Results. The smaller value was used because collicular and geniculate neurons receive input from 10–20 RGCs at the time of eye opening [75],[76]. In summary, Eq. 8 was designed to reduce the probability of a dendrite accepting a synapse if it was at or above its target firing rate, if there were too many synapses on the dendrite, or if there were too many synapses on the dendrite from the same presynaptic neuron.

Synapse resources in synapse 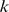 between presynaptic neuron 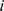 and postsynaptic neuron 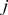 are represented by 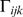. Upon formation, each synapse started with an initial level of resources, 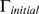, and could achieve a maximum of 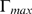. On each postsynaptic spike, trophic factor was released to the presynaptic terminal, where it was received and relayed to the axon. The axon, in turn, delivered synapse resources back to local synapses based on the amount of trophic factor present. The amount of synapse resources delivered to synapse 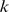 between presynaptic neuron 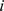 and postsynaptic neuron 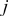 was:
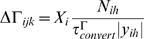
(9)where 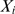 was the trophic factor to synapse resource exchange rate for neuron 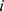 (Eq. 15), 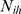 was the trophic factor present in the axon segment (Eq. 11), 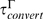 was the time constant regulating conversion of trophic factor to synapse resources, and 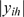 was the number of synapses residing on axon segment 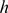. In other words, a set amount of trophic factor in each axon segment was converted to synapse resources and was distributed among all synapses on the segment. Eq. 9 was updated every 500 ms. On each presynaptic spike, synapse resources were decremented by a normal random value near unity (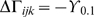). As discussed in Results, a modification of the model was examined where synapses acted independently and trophic factor received by synapses was converted to synapse resources at the level of individual synapses (i.e., 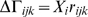). Both approaches resulted in qualitatively similar development (Fig. 4*E*).

#### Trophic factors

Trophic factors were released by the postsynaptic terminal in synapses where a postsynaptic spike followed a spike in the presynaptic cell within tens of milliseconds, whereafter it was taken up by the presynaptic terminal. The trophic factor, 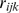, received by the presynaptic terminal of synapse 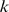 between presynaptic cell 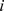 and postsynaptic cell 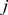 occurred after every spike in the postsynaptic cell and was:
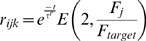
(10)where 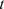 was the interval since the most recent presynaptic spike, 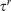 was a time constant governing the time window of trophic factor release, 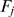 was the firing rate of postsynaptic neuron 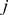 and 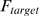 was the target firing rate of collicular neurons.

Trophic factor release was considered to be restricted to the synaptic terminal [13],[51], and trophic factor received by the synapse was relayed to the axon. In exchange, the axon provided a proportional amount of synapse resources to local synapses, based on an exchange rate set by the soma. The trophic factor in an axon diffused between adjacent segments and influenced the growth of each axon segment. The amount of trophic factor 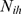 in axon segment 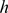 of neuron 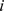 was calculated using:
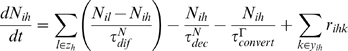
(11)where 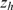 was the set of all axon segments connected to segment 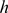, 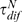 was the constant regulating diffusion between connected axon segments, 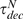 was the decay constant, 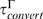 was the time constant regulating conversion of trophic factor to synapse resources, and 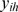 was the set of all synapses residing on axon segment 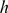. Trophic factor, 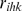, accumulated between each update of Eq. 11 (every 500 ms) and was reset to zero after each update.

#### Molecular guidance cues

Axon growth and synapse generation were influenced by the relative chemoaffinity of an axon segment for its surroundings. The chemoaffinity score of each axon segment was calculated by summing its approximated response from interactions with both ephrin-A/EphA and ephrin-B/EphB gradients (Eq. 2). These gradients are often composed of several different members of the same molecular families [43],[66]. For simplicity, the average response of all members of the same family are represented as a single gradient in the model. While “A” gradients were assumed to have a repulsive affect and “B” gradients were assumed to be growth promoting, the important functional behavior represented was that the chemoaffinity score was maximal near the retinotopically correct area of the colliculus and it decayed with distance, and Eqs. 12 and 13 approximate this. The mechanism implemented here resembles axon growth along the “A” gradients as described by [68], and a mechanism similar to a servomechanism, e.g., [77], along the “B” gradient axis. The chemoaffinity score 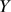 of axon segment 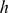 from RGC 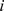 was:
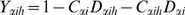
(12)

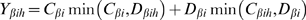
(13)where 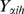 and 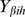 were the chemoaffinity scores for ephrin-A/EphA and ephrin-B/EphB gradients, respectively. The representations of 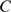 and 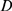 are as shown in shown in Fig. 10*A*, with 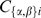 and 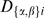 representing the chemoaffinity expression on all axon segments from neuron 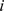, and 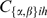 and 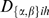 representing the chemoaffinity expression on collicular neurons at the location of segment 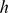 in the colliculus.

Disabling chemoaffinity was accomplished by setting all 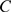 and 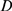 to zero for all cells. The results of ephrin knock-in experiments (e.g., [66],[78]) and of computational studies (e.g., [21],[66],[79],[80]) were not addressed in the present study, although a previous version of the present model [74] did briefly consider them. Analysis of the present model suggests that a homeostatic mechanisms to attract axons and synapses to underactive collicular neurons is required to replicate the results of experiments which manipulate ephrin expression. One such homeostatic mechanism is if underactive collicular neurons constitutively release growth factors to induce local axon and synapse growth [74]. Growth factor release was not included in the present model as it did not qualitatively affect development when molecular markers guided axons to the vicinities of their retinotopically correct termination zones (unpublished results).

#### Correlated retinal activity (retinal waves)

Spontaneous retinal activity was generated using a phenomenological model of retinal waves [30], which was based on a network of recurrently connected, spontaneously active cholinergic amacrine cells. This retinal wave model simulated spatiotemporal patterns of activity but not spiking patterns. To convert these patterns into spiking behavior, it was extended through representation of RGCs (Fig. 10*B*) which bursted when wave activity was present in their location in the retina. RGC intracellular potentials were considered to exceed threshold when the simulated 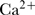 imaging signal (variable 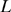, Eq. 4, in [30]) exceeded the detection threshold (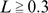). In the case of simulated 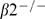 waves, the wave detection threshold was 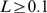 because the simulated calcium response was much weaker. Each RGC started bursting when the 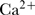 signal exceeded this threshold at the location in the retina corresponding to the nearest amacrine cell. To minimize artifacts caused by the poor spatial and temporal resolution of the simulated 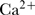 imaging, each RGC maintained its own activation threshold (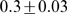, Gaussian distribution) that was recalculated after every burst and the burst start time was shifted 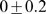 sec (Gaussian distribution). The mean burst frequency for each RGC was 20 Hz (Poisson distribution, with 3 ms refractory period) unless otherwise noted. The duration of each RGC burst was 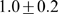 sec (Gaussian distribution), producing a spike cross-correlogram with a half-height width of near 1 sec, similar to that reported in P0 ferret [28], an age which corresponds to the early stage of acetylcholine mediated waves [5]. The spatiotemporal properties of the wave patterns used in this study, and the parameters used to generate them, are in Table 3. Retinal waves were generated on a 3.6 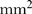 retina (as in [30]) and activity from the central section of the simulated retina was used to drive RGC activity in this study.

**Table 3:** Retinal wave generation.

Description	IWI (sec)	Velocity ( 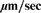 )	Size ( 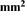 )	Avg. freq. (Hz)	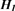	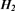	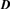	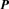	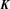	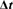 (sec)
Control (P2–P4 ferret)	94.2	180	0.161	0.21	4.0	0.75	1.3	35.0	0.25	0.025
High velocity	86.7	466	0.166	0.21	4.0	0.6	0.5	28.0	0.1	0.025
Low velocity	93.7	112	0.163	0.21	4.0	0.85	2.3	35.0	0.35	0.025
Short IWI	45.1	175	0.172	0.41	4.5	0.80	1.4	18.0	0.25	0.025
Long IWI	202	178	0.152	0.09	4.0	0.75	1.3	75.0	0.25	0.025
Small waves	87.9	181	0.101	0.22	3.0	1.25	1.4	28	0.25	0.025
Large waves	87.5	176	0.428	0.22	4.0	0.4	1.3	40	0.25	0.025
Simulated 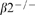	72.0	2,460	1.63	0.26	2.0	0.1	0.1	20.0	0.02	0.005

A previously published retinal wave model [30] was used to produce the patterns of retinal activity used in this study. Ferret retinal wave patterns served as control values. Variations from this control behavior were used to examine the sensitivity of development to particular spatiotemporal patterns of activity. The parameters 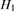, 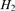, 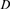, 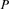, 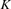 and 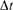 correspond to parameters of the same name as described originally [30]. RGC activity was pre-computed for each of the simulations. 24 hours of wave data was pre-computed (12 hours for short IWI and 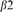) and this sequence of activity was repeated as many times as necessary to provide continuous patterns of retinal activity. The parameters and wave statistics for 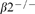 waves, particularly the extremely high wave velocity, were selected to produce correlations as reported experimentally [26].

#### Homeostatic controls

Many homeostatic mechanisms were governed by how much a neuron was above or below its target firing rate (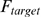). When a collicular neuron was below its target firing rate, it was more likely to accept new synaptic connections and thereby increase total excitation, whereas when a neuron was above its target firing rate, it reduced the amount of trophic feedback to innervating synapses to induce synaptic retraction and thereby decrease excitation. Each neuron also regulated the strength of its innervating synapses based on changes in its firing rate [56]. The homeostatic scaling factor for synaptic strength, 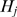, was:
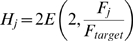
(14)The multiplicative constant 2 was used to scale the output of 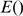 so 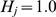 when 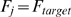. Analysis of the model indicated that homeostatic scaling of synaptic weights was not required for refinement, as disabling this mechanism (i.e., 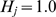) resulted in only minor deficits to refinement (group RF +2%; PF +1%), suggesting that synaptic scaling might be a redundant homeostatic mechanism, or one that, like constitutive growth factor release (see *Molecular guidance cues*, above), might be necessary for behaviors not examined in the present study.

The number of axonal synapses was also subjected to homeostatic controls. The probability of new synapse creation depended on how many synapses were already on a particular axon (Eq. 7). Additionally, each neuron regulated the availability of synapse resources based on how many synapses were present on its axon, reducing the amount of resources required for synapse survival on a sparsely populated axon and increasing the amount required with increasing synapse count. This was accomplished through use of an “exchange rate”, 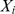, governing how many synapse resources were available for a given amount of trophic feedback received by a synapse. Specifically:
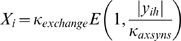
(15)where 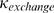 was the base exchange rate for a neuron with 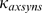 axonal synapses, and 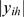 was the number of axonal synapses. Setting an exchange rate based on total (or approximate) axonal synapse count allowed synapse resources to be managed by the soma, but required minimal information exchange between the soma and the axon. This resulted in the necessary information for synapse survival and retraction to be had at the level of the individual synapse.

#### Neuron model

The model used integrate and fire neurons that were modified to approximate the effects of dendritic growth and homeostatic regulation of firing rate. Neural excitation levels were recalculated every millisecond and each neuron produced an action potential when its level of excitation exceeded threshold, whereafter its excitation level was reset to zero.

The resting conductance of the neuron 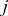, 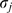, started at a base value of 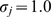, and with time increased towards its maximum value, 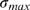, representing the relative conductance of a mature neuron. The increase in conductance was assumed to occur largely from dendritic growth. Model dendrites started small and grew with time, with growth measured by the electrical size of the dendrite. For computational convenience, dendrites were assumed to have a constant arborization radius and physical growth was considered to result from increased arbor complexity. A neuron's dendrite grew when the cell was firing near or above its target firing rate, as this was considered to imply sufficient synaptic input to activate the requisite growth mediating pathways (e.g., [81],[82]). The conductance of the neuron changed according to:
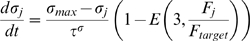
(16)where 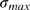 was the maximum conductance, 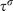 was the growth time constant, 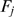 was average firing rate of neuron 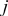, and 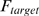 was the target firing rate. This equation was recalculated at every 500 ms.

The excitation level, 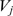, for postsynaptic neuron 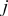 was calculated using:
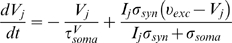
(17)where 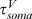 is the soma decay constant (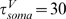 ms), 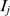 represents the total synaptic input, 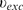 is the reversal potential of excitatory synapses (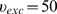) and 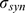 is the conductance of an individual synapse. When 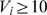, an action potential occurred and 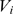 was reset to zero. 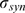 was calculated to produce a specific peak rate of depolarization in the soma of the target neuron by activation of a single synapse. A non-potentiated synapse would produce an EPSP of 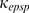 (see Table 1) in the soma of an immature dendrite (i.e. 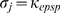).

Excitation to neuron 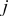 from synaptic input, 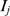, was calculated using:
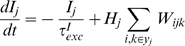
(18)where 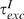 is the time constant for excitatory input, 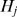 is a homeostatic scaling factor (Eq. 14), 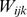 is the strength of synapse 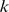 between presynaptic neuron 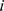 and postsynaptic neuron 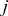 (Eq. 20), and 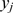 is the set of all dendritic synapses. Eqs. 17 and 18 were recalculated every millisecond.

#### STDP

The implementation described here is derived from the STDP triplet model of [8], which was modified to observe saturation of plasticity, where maximal plasticity was realized after a finite number of spike pairs [7],[58]. As described by [8], each presynaptic neuron 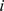 and postsynaptic neuron 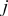 had an efficacy, 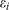 and 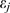 respectively, that depended only on the interval from the present spike to the immediately preceding spike in the same neuron. Efficacy was set to zero immediately after a spike and exponentially recovered to 1.0:
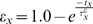
(19)for 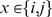. The interval since the preceding spike in the same neuron was represented by 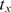. The efficacy time constant 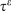 was different for pre and postsynaptic neurons: 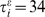 ms and 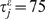 ms [8].

Except as otherwise noted, all non-potentiated synapses in the model were considered to have a unitary base strength and potentiation and depression was relative to this unitary strength. Weight changes to each synapse were additive, such that 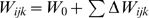, with 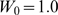 being the starting level of potentiation. The weight change realized by a synapse, 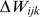, after a pre- or postsynaptic spike was:
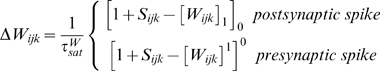
(20)where 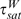 was the time constant regulating how quickly the synapse approached its saturation plasticity level and 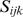 was the saturation level of plasticity, a value based on the interval since the most recent spike in the opposite neuron. The inner brackets in Eq. 20 restrict plasticity changes in order to limit a weakly potentiating spike pair in a previously depressed synapse to the magnitude as would occur in a non-depressed synapse (i.e. a weakly potentiating spike pair was limited to small magnitude changes). This magnitude limiting mechanism was to prevent an extremely weakly potentiating spike pair (e.g. separated by 75 ms) from producing anything other than extremely weak potentiation in an already depressed synapse. The outer brackets prevent spike pairings that saturate at lower magnitude potentiation from weakening an already potentiated synapse. The same principles described here applied for both potentiation and depression. The value 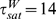 spikes was a constant governing how quickly synaptic weights would approach their saturation level and was approximated using the observed rate of STDP saturation [7].

The saturating level of plasticity change, 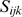, for a given spike pair, of synapse 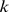 between presynaptic neuron 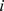 and postsynaptic neuron 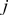, was:
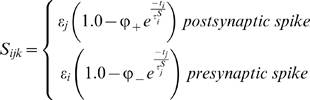
(21)where 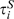 and 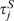 were the time constants governing the time window for STDP sensitivity (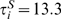 ms and 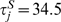 ms), 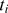 and 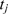 were the times since the most recent spikes in the pre- or postsynaptic neuron, respectively, and 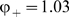 and 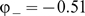 were the peak magnitudes of potentiation and depression, respectively. Values for 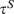 and 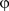 are from [8].

## Supporting Information

Video S1 Axon development in the colliculus. Axons from 5 retinal locations are shown over 120 hours of simulated development. The first 60 hours of growth are mediated by molecular guidance cues only, allowing axons to extend to near their retinotopically correct locations. During the second 60 hours of development, activity-dependent mechanisms contribute to axon growth. Axons quickly refine after the onset of activity-dependent mechanisms.
(4.95 MB AVI)
